# The College of Nursing Election

**Published:** 1918-03-16

**Authors:** 


### The College of Nursing Election.

### Preliminary Instructions to Members.

MEMBERS of the College need not wait until they receive their nomination papers before deciding whom they wish to nominate and vote for. Nurses who are members of co-operations or who belong to the staffs of hospitals or Poor-Law infirmaries will know some of their number who are especially suited to express the ideals and needs of the profession. These are the nurses whose consent for nomination their colleagues should at once secure. Then, when the nomination papers are delivered, no time need be lost.

Two members of the College, one as a proposer and another as a seconder, will nominate the desired candidate, whose consent to be nominated will also be indicated on the nomination form. There will be English and Welsh sections, Scotch and Irish, determined by the postal addresses of members. The proposer and seconder must be of the same section to nominate a representative for that section, but a member's vote may be distributed over any or all sections. The candidate for election need not be a member of the College, but two members must take part in the nomination of any candidate, and the elected will be those who have received the greatest number of votes of members. This is a constitution which nurses should hold truly democratic, and others will not fail to appreciate.

